# Clinical–psychosocial archetypes predict short-term outcomes in inflammatory arthritis: an unsupervised segmentation study

**DOI:** 10.1007/s10067-026-08186-9

**Published:** 2026-05-28

**Authors:** Dmytro Fedkov, Danylo Yevstifeiev, Oleg Iaremenko, Daria Koliadenko, Liubov Petelytska, Christine Peine, Felix Lang, Abdullah Khalil, Türker Kurt, Stefan Vordenbäumen

**Affiliations:** 1 Department of Internal Medicine #3, Bogomolets National Medical University, 13 Blvd Shevchenka, Kyiv, 01032 Ukraine; 2 Medical Center Medical Clinic Blagomed LLC, Kyiv, Ukraine; 3 Midaia GmbH, Heidelberg, Germany; 4 Department Rheumatology, . Elisabeth-Hospital Meerbusch-Lank, Hauptstr. 74-76, 40668 Meerbusch, Germany; 5 Department of Rheumatology, University Hospital Düsseldorf, Medical Faculty of Heinrich Heine University, Düsseldorf, Germany; 6 Hiller Research Center, University Hospital Düsseldorf, Medical Faculty of Heinrich Heine University, Düsseldorf, Germany

**Keywords:** Digital health, Psoriatic arthritis, Rheumatoid arthritis, Spondyloarthritis

## Abstract

**Background:**

Heterogeneity in inflammatory arthritis (IA) outcomes limits the effectiveness of non-individualized treatment approaches, and digital health platforms can capture psychosocial and behavioral signals that may stratify responses beyond diagnosis or baseline severity.

**Objective:**

To identify clinically interpretable patient clusters and evaluate their associations with 12-week outcomes in IA, testing whether cluster membership adds information beyond demographics, diagnosis, and baseline symptom burden.

**Methods:**

We retrospectively analyzed the use of a CE-certified rheumatology application among adult patients. Baseline clinical and psychosocial variables (Patient’s Global Assessment of Disease Activity (PGADA) and Patient’s Global Assessment of Pain Intensity, sleep quality, social support, distress, fatigue, activity, diet/fasting) were winsorized (1st/99th), z-scaled, and imputed by median/mode for features only; outcomes were complete-case. Unsupervised k-means (k = 5) was selected based on silhouette, gap, and consensus diagnostics. The primary validation outcome was remission (12-week PGADA ≤ 20 mm for patients with baseline PGADA ≥ 40 mm), with distributional changes in PGADA and percentage change as secondary endpoints.

**Results:**

Among 2,924 patients, five clusters were identified (size range 17.7–22.9% of the cohort). The 12-week remission rate was 7.0%, with the "resilience" profile (characterized by better sleep, stronger social support, and lower distress) showing the highest probability of remission and the most favourable PGADA distribution. In contrast, distress-dominant clusters (characterized by poor sleep and weak support) showed the lowest remission rates and minimal improvement. The median ΔPGADA% was 8.3% (IQR − 8.2% to 32.0%). In adjusted analyses, the cluster signal persisted beyond baseline severity; percentage-change estimates were attenuated for clusters with lower baseline PGADA.

**Conclusion:**

Cluster-level phenotypes derived from routinely collected app data align with short-term clinical outcomes, highlighting sleep, social support, and distress as modifiable factors that may influence short-term outcomes. Programs should emphasize the quality of activity and recovery (not just volume), particularly for patients with high distress and poor sleep. Future work should evaluate cluster-informed, multicomponent interventions in prospective studies.

**Table:** 

**Key Points** • *Clinical–psychosocial archetypes derived from routinely collected app data (symptoms, sleep, social support, distress, lifestyle) were strongly associated with 12-week remission and PGADA change, beyond diagnosis and baseline severity.* • *Distress-dominant archetypes with poor sleep and weak social support had the lowest remission rates and minimal improvement, indicating that unaddressed psychological burden and sleep problems can blunt the benefits of otherwise appropriate pharmacological care.* • *Resilient archetypes, with better sleep, stronger social support, lower distress, and healthier lifestyle patterns, showed the most favourable outcomes, supporting a stratified care model in which digital tools help identify high-risk patients and prioritise targeted behavioral, psychosocial, and recovery-focused interventions rather than simply prescribing more physical activity.*

**Supplementary Information:**

The online version contains supplementary material available at 10.1007/s10067-026-08186-9.

## Introduction

Inflammatory arthritides (IA), including rheumatoid arthritis (RA), psoriatic arthritis (PsA), and spondyloarthritis (SpA), are characterised by substantial clinical and pathobiological heterogeneity [1]. This diversity poses a significant challenge in modern rheumatology, leading to considerable variability in patient treatment responses. Despite therapeutic advances, a large proportion of patients fail to achieve desired clinical targets: approximately 40% do not respond to individual disease-modifying antirheumatic drugs [2, 3], and only 20–30% attain low disease activity [4]. This "ceiling effect" in therapeutic efficacy is primarily a consequence of a persistent "trial and error" approach, driven by a lack of robust predictive biomarkers that could align treatments with individual patient pathobiology [1].

In response to these challenges, rheumatology is undergoing a paradigm shift from uniform treatment strategies toward stratified and personalized medicine [5, 6]. Artificial intelligence and machine learning are pivotal in this transformation, offering powerful tools to analyze large-scale, multidimensional data and identify clinically meaningful patient subgroups [5, 7]. Previous research has successfully applied clustering methods to stratify patients with IA using diverse data types, including synovial histological and molecular signatures [8, 9], genetic markers [10], and clinical trial data [5, 7]. These approaches have demonstrated the potential to predict therapeutic response and optimize clinical decision-making [6]. However, the majority of these studies have focused predominantly on biological and clinical variables, often overlooking a broader spectrum of factors that influence disease outcomes.

A growing body of evidence suggests that psychosocial factors, including psychological distress, sleep quality, and social support, are significant determinants of clinical outcomes in IA [11]. Psychological distress, including depression and anxiety, is not only prevalent among patients with IA [11, 12], but is also strongly associated with higher disease activity, increased pain, poorer treatment response, and even increased mortality [12, 13]. The relationship is bidirectional: chronic inflammation can contribute to depressive symptoms via its effects on the central nervous system, while psychological stress can amplify inflammatory processes through neuroendocrine pathways, such as the hypothalamic–pituitary–adrenal axis [11, 12]. Similarly, sleep disturbances, a common complaint in SpA and PsA [14, 15], are correlated with higher levels of pain, depression, and diminished quality of life [14]. Conversely, resilience factors, such as high-quality sleep and strong social support, are associated with better treatment outcomes, underscoring their importance in a comprehensive care model [16]. Furthermore, demographic factors, such as sex, cannot be ignored, as studies indicate sex-based differences in response to biologic therapies in PsA [17].

The role of lifestyle factors, particularly physical activity, in managing IA is complex. While exercise is broadly recommended to improve physical function and reduce cardiovascular risk [18], its effects can be paradoxical [19]. An emerging concept of a "physical activity paradox" suggests that the benefits of exercise may be negated in patients with high levels of psychological distress or poor sleep quality [19, 20]. This indicates that addressing psychological and behavioral factors may be a prerequisite for fully unlocking the therapeutic potential of physical activity.

The integration of such multifaceted data into patient stratification models remains underexplored. Digital health technologies and mobile applications offer unprecedented opportunities to collect comprehensive, real-world data that capture not only clinical metrics but also psychosocial and behavioral patterns [10, 21]. The use of electronic patient-reported outcomes enables continuous monitoring, fostering patient-centric and hybrid models of care [21, 22]. Analysis of such rich datasets with unsupervised machine learning can uncover naturally occurring, clinically relevant patient phenotypes that transcend traditional diagnostic boundaries.

Therefore, in this study, we applied an unsupervised machine learning approach to a large, real-world cohort of patients with inflammatory arthritis using a digital therapeutic application. Our primary objective was to identify distinct clinical-psychosocial phenotypes at baseline. A secondary objective was to assess the clinical relevance of these derived archetypes by examining their association with behavioral engagement and clinical outcomes at 12 weeks. We hypothesized that this holistic, data-driven stratification would identify patient archetypes with distinct clinical trajectories, providing a foundation for developing more personalized care strategies.

## Methods

### Study design and patient cohort

This study was a retrospective analysis of a real-world cohort of 2,924 patients with physician-confirmed inflammatory arthritis, including RA, PsA, and SpA. Data were collected between January 2022 and June 2025 from users of the Mida Rheuma App (Midaia GmbH, Germany), a CE-certified medical device [23]. While the Digital Health Application facilitates continuous data entry, for this analysis, data were extracted at two key time points: baseline (at enrollment) and a 12-week follow-up. Inclusion criteria were: adult patients (≥ 18 years) with a diagnosis of RA, SpA, or PsA confirmed by a rheumatologist; consent to use data for research purposes; and availability of baseline data.

The primary objective of this study was to apply a data-driven, unsupervised machine learning approach to identify distinct clinical–psychosocial phenotypes at baseline. A secondary objective was to assess the clinical relevance of these derived archetypes by examining their association with behavioral engagement and clinical outcomes at 12 weeks. This study focuses on unsupervised patient segmentation to identify naturally occurring archetypes. Analyses of Remission and percentage change in Patient Global Assessment of Disease Activity (PGADA) by cluster were prespecified to assess the external face validity of these archetypes and are interpreted as associations supporting the clinical relevance of the segmentation rather than as predictive modelling. Remission (PGADA ≤ 20 mm) was evaluated only in patients with baseline PGADA ≥ 40 mm. We purposely did not develop or assess individual-level prognostic models in this study.

The study was conducted in accordance with the ethical principles outlined in the Declaration of Helsinki (October 2024 revision) and the Good Clinical Practice guidelines. It was approved by the Local Ethic Committee of Medical Center Medical Clinic Blagomed LLC (protocol #107/5, dated 13 Aug 2022). All patients provided informed consent before using the Mida Rheuma App.

### Data source and digital therapeutic intervention

The Mida Rheuma App is designed to support patient self-management and facilitate multimodal lifestyle counselling. Key features include the tracking of disease activity, patient-reported outcomes, medication intake, and lifestyle parameters. Based on this input, the application delivers a multimodal, guideline-based intervention through personalized "action plans". These plans are designed to improve health literacy and encourage self-management in domains such as nutrition, exercise, and mental well-being, consistent with EULAR recommendations for patient care.

### Statistical analysis

All variables for the clustering model were selected from the baseline assessment to ensure that the derived phenotypes could be used for prognostication at the point of care. The selection of variables was guided by a data completeness analysis to maximize the use of observed data and minimize reliance on imputation, while maintaining clinical interpretability. Variables with more than 30% missingness were generally excluded. For retained variables, missing entries were imputed with the median (continuous) or mode (binary/categorical) computed from the baseline cohort; outcome analyses were performed on complete cases.

Several instruments measuring disease activity and functional status were evaluated. The composite Routine Assessment of Patient Index Data 3 (RAPID3Baseline) [24] score was not selected for the primary model due to substantial missingness (34.2%). In contrast, its component measures, the PGADA [25], 0–100 mm, and Patient's Global Assessment of Pain Intensity (PPAIN) [26], 0–100 mm on a visual analogue scale, demonstrated much higher data completeness (12.3% missingness for both). Consequently, to construct a model based on more robust, directly observed data, these more complete component variables were chosen. The final set of variables included demographics and disease history (age, sex, diagnosis, disease duration), clinical burden (PGADA, PPAIN, fatigue via Brief Fatigue Inventory (BFI) Baseline) [27], psychological status via Patient Health Questionnaire-4 (PHQ4 Score Baseline) [28, 29], and lifestyle/anthropometric data (Body Mass Index, and engineered composite indices for Diet Quality, Activity, Sleep Quality, Fasting Status, and Social Support). The composite indices were study-specific operationalizations derived from app-based variables rather than previously validated standalone composite instruments. They were designed to summarize conceptually relevant behavioral and psychosocial domains for digital phenotyping and clustering, and should therefore be interpreted as pragmatic app-derived features rather than validated scales.

To create these composite indices, several variables were engineered. The Physical Activity Score was the sum of self-reported frequency scores (0–4) across five exercise domains (strenuous, moderate, strength, flexibility, and sensorimotor), yielding a score from 0 to 20. The Social Support Score was the sum of scores from two items assessing support from "Family" and "Friends", each rated on a 0–10 scale (range 0–20). The Diet Quality Score was constructed as an additive index reflecting adherence to an anti-inflammatory diet, where + 1 point was awarded for beneficial habits and −1 point was subtracted for detrimental ones, based on self-reported frequency (participants indicated how often they typically consumed the foods, using a 5-level scale from 1 ("never") to 5 ("4 + times per day/week," product-dependent)). Beneficial habits included frequent consumption of vegetables (score ≥ 4), fruits (score ≥ 4), and oily fish (score ≥ 3). Detrimental habits included frequent consumption of red meat (score ≥ 4), sausage products (score ≥ 4), sugar (score ≥ 4), soft drinks (score ≥ 3), and frequent addition of salt (score ≥ 4). The final score represents the sum of these points (range −5 to + 3), with higher values indicating a healthier diet [30, 31]. Similarly, the Sleep Quality Score was derived by subtracting the count of reported sleep problems (each coded as 1 if present: "Can't Sleep", "Wakes Up at Night", "Wakes Up Too Early", "Nightmares") from a general sleep satisfaction score (rated 0–4), resulting in a scale from −4 to + 4. Finally, Fasting Status was converted into a binary variable (0 = no prior experience, 1 = prior experience).

Prior to analysis, the data underwent several preprocessing steps. To reduce the leverage of extreme outliers, all continuous variables were winsorized at the 1 st and 99th percentiles. Categorical variables (sex, diagnosis) were converted to a numerical format using one-hot encoding. Finally, all variables were standardized to a mean of 0 and a standard deviation of 1 (z-scaling) to ensure their equal contribution to the clustering algorithm.

To derive robust clinical phenotypes, we employed a multi-step approach. First, Principal Component Analysis was performed on the preprocessed dataset to reduce noise and multicollinearity. Based on the scree plot ("Elbow method") and cumulative variance explained, the first five principal components were retained for subsequent analysis. These five components explained 65.0% of the cumulative variance. Although this proportion is moderate, it was considered appropriate for heterogeneous real-world clinical data combining objective variables and subjective patient-reported outcomes. Retaining additional components provided only marginal gains in explained variance while increasing the risk of introducing noise into the clustering algorithm.

Second, consensus k-means clustering was performed on these retained components using the "ConsensusClusterPlus" package in R. This method provides a robust estimation of cluster stability by repeatedly subsampling the data (80% of patients, 80% of features) over 1000 repetitions. We evaluated solutions for a range of cluster numbers from k = 2 to k = 6. The optimal number of clusters was determined by evaluating multiple stability metrics. Visual inspection of the cumulative distribution function curves and the delta-area plot revealed an apparent elbow at k = 5 (Supplementary Figure S1). At k = 5, the consensus matrix exhibited well-defined, high-consensus blocks with minimal off-diagonal agreement. In contrast, the k = 4 solution retained heterogeneous within-cluster structure, and the k = 6 solution produced fragmented blocks and unstable membership in the tracking plot, suggesting over-partitioning (Supplementary Figure S2). This qualitative assessment was quantitatively supported by the Proportion of Ambiguous Clustering (PAC): the k = 5 solution exhibited the lowest PAC value (0.168) compared to solutions with k = 4 (0.367), k = 6 (0.182), k = 3 (0.564), and k = 2 (0.675), providing robust quantitative evidence for its selection. We therefore selected k = 5 for all downstream analyses.

As medication data were unavailable, we conducted an internal robustness check to assess whether an uncontrolled baseline inflammatory burden could substantially influence the clustering structure. To do so, we explored an alternative clustering solution that included baseline disease activity as an additional feature. This sensitivity analysis allowed us to assess whether the psychosocial–behavioral archetypes identified in the main model were robust to potential confounding by disease severity. Because the resulting clusters were dominated by symptom-severity patterns and addressed a different research question, they were excluded from the final analysis (Supplementary Table S1).

The derived clusters were externally validated using the primary outcome of Remission at 12 weeks. Specifically, remission was defined as achieving a PGADA score of ≤ 20 (on a 0–100 mm scale) by week 12 among patients with a baseline PGADA score of ≥ 40. The selection of PGADA as the primary outcome anchor is methodologically grounded in its concordance with ACR/EULAR guidelines, where omitting this core component results in poorer prognostic performance for functional and radiographic outcomes. Our specific remission threshold of PGADA ≤ 20 aligns with updated, empirically tested criteria that optimize the identification of patients in this state without significantly compromising these long-term outcomes [32]. Furthermore, because our cohort includes patients with both RA and SpA, PGADA provides essential cross-disease comparability, serving as a standardized anchor by being a validated component of key indices across these diagnoses (e.g., the Ankylosing Spondylitis Disease Activity Score and the Disease Activity in Psoriatic Arthritis) [33–35]. From a content validity perspective, PGADA offers a more holistic reflection of the disease's overall impact by capturing not only pain but also stiffness and fatigue, which is crucial for avoiding the misclassification risk associated with non-inflammatory pain drivers, such as central sensitization [34]. Finally, this choice was supported by an essential analytical advantage: it prevents measurement coupling, since patient-reported pain was a feature in the baseline clustering model. The secondary outcome was the percentage change in PGADA (ΔPGADA%) from baseline to week 12.

Baseline characteristics were summarized for the overall cohort and stratified by cluster, using means and standard deviations for continuous variables and counts/percentages for categorical variables. Differences in clinical outcomes across clusters were evaluated using the Kruskal–Wallis test for continuous variables and the Pearson's Chi-squared test (χ^2^) for categorical variables, with post-hoc pairwise comparisons using a Z-test for two proportions with a Bonferroni correction. Means ± SD were also reported for interpretability.

To test whether cluster-level associations were independent of key baseline confounders, we fitted multivariable regression models. The association between cluster membership and Remission achievement was estimated using logistic regression, reporting odds ratios (ORs) and 95% confidence intervals (CIs). The association with ΔPGADA% was assessed using linear regression, reporting beta coefficients (β) and 95% CIs. Both models were adjusted for age, sex, diagnosis, disease duration, baseline pain, and baseline PGADA.

All statistical analyses were performed using R (Version 4.5.1, R Foundation for Statistical Computing, Vienna, Austria), and a two-sided *p*-value of < 0.05 was considered statistically significant.

## Results

The final analytical cohort for baseline clustering comprised 2,924 patients with a physician-confirmed diagnosis of IA. Of this initial cohort, 1,996 patients provided PGADA data at the 12-week follow-up. For the primary outcome analysis (remission), a subset of 684 patients was evaluated, meeting criteria for both follow-up data and a baseline PGADA ≥ 40 mm.

The mean age of the whole baseline cohort (*N* = 2,924) was 45.0 ± 13.0 years, 76% were female, and the mean disease duration was 8.4 ± 9.5 years. The cohort was diagnostically diverse, RA being the most common diagnosis (55%), followed by SpA (24%) and PsA (12%).

Overall, within the remission analysis subset (*N* = 684), the 12-week remission rate was 7.0% (PGADA ≤ 20 mm). The overall median ΔPGADA% in this subset was 8.3% (IQR − 8.2% to 32.0%).

**Fig. 1:**
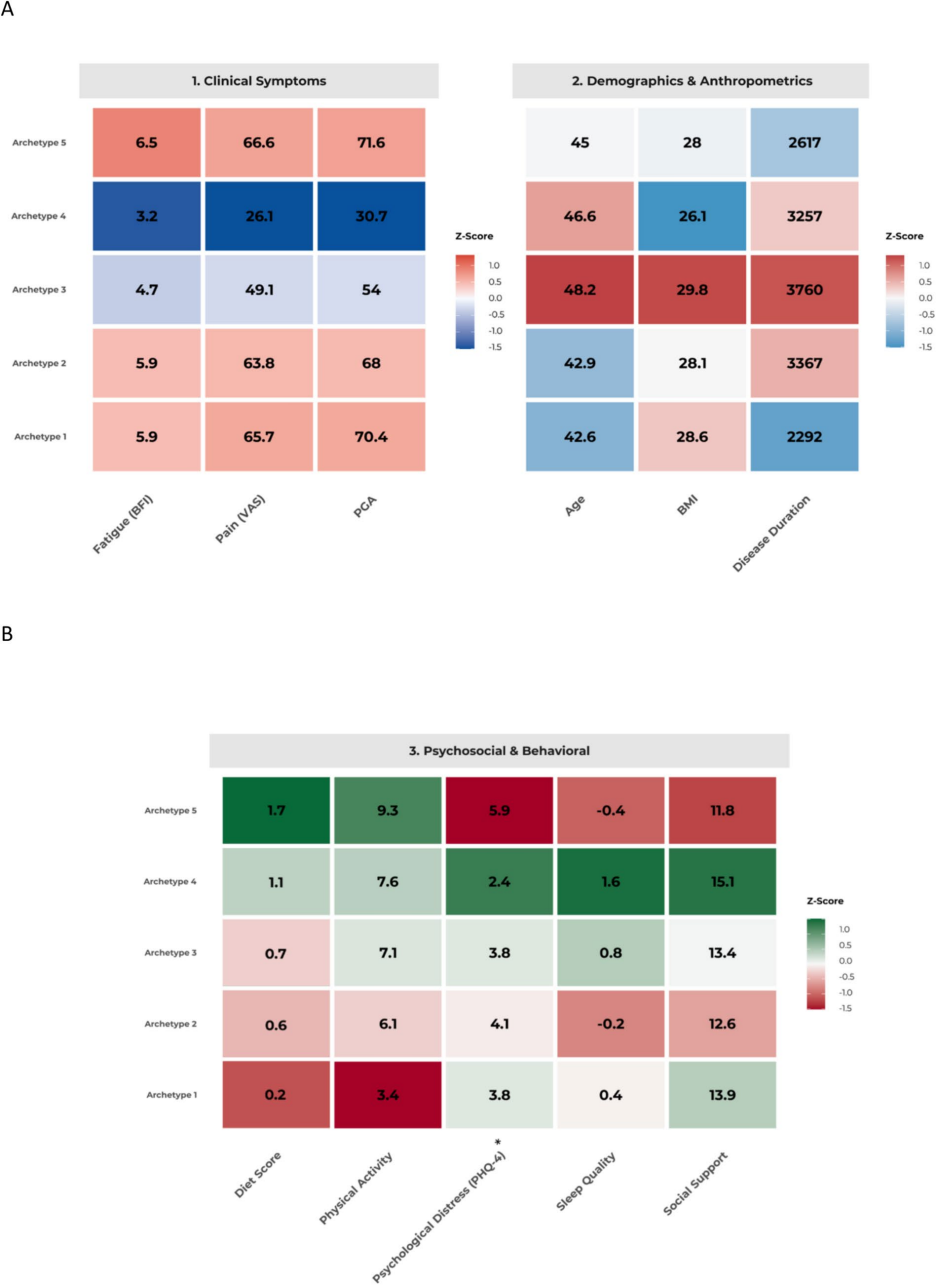
Baseline archetypes. Heatmap of baseline archetype profiles, presented in two panels. Each cell displays the mean value of a characteristic for that archetype, while the cell colour is determined by the standardized Z-score for that variable. Panel (**A**) displays "1. Clinical Symptoms" and "2. Demographics & Anthropometrics" using a blue-red palette. In this panel, red indicates a high Z-score, and blue indicates a low Z-score. Panel (**B**) displays "3. Psychosocial & Behavioral" using a green–red palette. In this panel, green indicates a high Z-score, and red indicates a low Z-score. * To ensure an intuitive colour scheme, colours for PHQ-4 have been inverted. Therefore, in this panel, red consistently represents an unfavourable attribute, while green represents a favourable attribute

Unsupervised clustering of the baseline data, using principal components, followed by clustering, identified five distinct and clinically interpretable patient archetypes. The distribution of all baseline characteristics differed significantly across the five clusters (*p* < 0.001 for all variables), confirming a robust and meaningful segmentation (Table 1**, **Fig. 1). Based on their defining features, the archetypes were profiled as follows.

- **Cluster 1, "RA-Female, Low Physical Activity/Pain-Dominant" (N = 669)**, consisted almost exclusively of female patients (98%) with RA (99%). This archetype was characterized by a high burden of pain (66 ± 18) and high disease activity (70 ± 16), accompanied by significant fatigue. Notably, this group reported the lowest physical activity levels (3.4 ± 4.1).
- **Cluster 2, "SpA-Female, Low-Support/High-Symptom" (N = 517)**, was predominantly composed of female patients (98%) with a high proportion of SpA (75%). They presented with high symptom severity across PGADA, pain, and fatigue, comparable to Cluster 1. A key distinguishing feature was having the lowest level of social support (12.6 ± 4.4).
- **Cluster 3, "Male, Long-Duration/Moderate-Symptom" (N = 668)**, was uniquely composed of male patients (96%) and had the most extended mean disease duration (10.3 ± 11.78 years). Their clinical symptoms, including PGADA, pain, and fatigue, were moderate and hovered around the cohort average.
- **Cluster 4, "Female, High-Resilience/Low-Symptom" (N = 531)**, represented the most favourable profile. This group had the lowest levels of pain (26 ± 18), disease activity (31 ± 20), fatigue (3.18 ± 1.7), and psychological distress (2.4 ± 1.8). Concurrently, they reported the best sleep quality and the highest social support (15.1 ± 3.7), positioning them as a highly resilient group.
- **Cluster 5, "Psychologically Distressed, Poor Sleep/High-Symptom" (N = 539)**, while also presenting with very high pain (67 ± 18) and PGADA (72 ± 17), was uniquely defined by the most severe psychological burden. They exhibited the highest levels of fatigue (6.46 ± 1.33), the highest psychological distress (5.9 ± 2.8), and the poorest sleep quality (−0.42 ± 1.81).

**Table 1:** Baseline characteristics of the five patient archetypes

	**Patient Archetype**						
**Characteristic**	**Overall** N = 2,924^1^	**1** N = 669^1^	**2** N = 517^1^	**3** N = 668^1^	**4** N = 531^1^	**5** N = 539^1^	***p*-value**^2^
Age (years)	45 ± 13 (19)	43 ± 13 (11)	43 ± 12 (6)	48 ± 14 (2)	47 ± 13 (0)	45 ± 13 (0)	< 0.001
Disease Duration (years)	8.4 ± 9.5 (619)	6.3 ± 6.7 (243)	9.2 ± 9.6 (159)	10.3 ± 11.8 (156)	8.9 ± 9.2 (59)	7.2 ± 8.5 (2)	< 0.001
**Sex**							< 0.001
Female	2,210 (75.6%)	655 (97.9%)ᵃ	506 (97.9%)ᵃ	0 (0%)ᵇ	519 (97.7%)ᵃ	530 (98.3%)ᵃ	
Male	663 (22.7%)	0 (0%)ᶜ	0 (0%)ᶜ	644 (96.4%)ᵃ	12 (2.3%)ᵇ	7 (1.3%)ᵇ	
Other	22 (0.8%)	0 (0%)ᵇ	0 (0%)ᵇ	20 (3.0%)ᵃ	0 (0%)ᵇ	2 (0.4%)ᵇ	
Undefined	29 (0.99%)	14 (2.1%)ᵃ	11 (2.1%)ᵃ	4 (0.6%)ᵃ	0 (0%)ᵇ	0 (0%)ᵇ	
**Diagnosis**							< 0.001
RA	1,618 (55%)	661 (98.8%)ᵃ	0 (0%)ᵈ	324 (49%)ᶜ	365 (68.7%)ᵇ	268 (49.7%)ᶜ	
SpA	705 (24%)	0 (0%)ᵉ	386 (75%)ᵃ	210 (31%)ᵇ	81 (15.3%)ᶜ	28 (5.2%)ᵈ	
PsA	364 (12%)	4 (0.6%)ᵈ	35 (6.8%)ᶜ	88 (13%)ᵇ	67 (12.6%)ᵇ	170 (31.5%)ᵃ	
RA and SpA	100 (3.4%)	1 (0.1%)ᶜ	66 (13%)ᵃ	19 (2.8%)ᵇ	8 (1.5%)ᵇ	6 (1.1%)ᵇ	
RA and PsA	65 (2.2%)	1 (0.1%)ᶜ	26 (5.0%)ᵃ	11 (1.6%)ᵇ	5 (0.9%)ᵇ	22 (4.1%)ᵃ	
SpA and PsA	48 (1.6%)	1 (0.1%)ᵇ	0 (0%)ᵇ	9 (1.3%)ᵇ	3 (0.6%)ᵇ	35 (6.5%)ᵃ	
RA and SpA and PsA	24 (0.8%)	1 (0.1%)ᵇ	4 (0.8%)ᵃ	7 (1.0%)ᵃ	2 (0.4%)ᵃ	10 (1.9%)ᵃ	
**Outcomes**							
PGADA	58 ± 25 (360)	70 ± 16 (166)	68 ± 18 (71)	54 ± 26 (115)	31 ± 20 (0)	72 ± 17 (8)	< 0.001
PPAIN	54 ± 26 (360)	66 ± 18 (166)	64 ± 19 (71)	49 ± 26 (115)	26 ± 18 (0)	67 ± 18 (8)	< 0.001
BFI	5.20 ± 1.96 (467)	5.87 ± 1.38 (194)	5.89 ± 1.44 (91)	4.73 ± 1.85 (148)	3.18 ± 1.70 (19)	6.46 ± 1.33 (15)	< 0.001
PHQ-4	4.1 ± 2.7 (1,039)	3.8 ± 2.1 (426)	4.1 ± 2.6 (232)	3.8 ± 2.4 (257)	2.4 ± 1.8 (107)	5.9 ± 2.8 (17)	< 0.001
BMI	28.1 ± 16.7 (600)	28.6 ± 27.2 (238)	28.1 ± 9.4 (115)	29.8 ± 21.1 (180)	26.1 ± 7.1 (44)	28.0 ± 9.8 (23)	< 0.001
Diet Quality Score	0.84 ± 1.26	0.23 ± 0.94	0.62 ± 1.22	0.71 ± 1.11	1.08 ± 1.14	1.70 ± 1.07	< 0.001
Activity Score	6.9 ± 4.7 (673)	3.4 ± 4.1 (275)	6.1 ± 4.9 (123)	7.1 ± 4.8 (205)	7.6 ± 4.5 (42)	9.3 ± 3.3 (28)	< 0.001
Sleep Quality Score	0.44 ± 1.92 (608)	0.40 ± 1.82 (259)	−0.18 ± 1.96 (116)	0.75 ± 1.84 (185)	1.58 ± 1.68 (35)	−0.42 ± 1.81 (13)	< 0.001
Fasting Experience, n (%), Missing	2,096 (93%), 665	381 (96%), 274	368 (93%), 122	448 (96%), 201	436 (89%), 42	463 (90%), 26	< 0.001
Social Support Score	13.4 ± 4.3 (680)	13.9 ± 4.0 (275)	12.6 ± 4.4 (124)	13.4 ± 4.2 (205)	15.1 ± 3.7 (46)	11.8 ± 4.3 (30)	< 0.001

^1^Data are presented as mean ± SD (Missing) for continuous variables and n (%) for categorical variables. Percentages for categorical variables are calculated within each cluster

^2^*p*-value represents the statistical difference between the five archetype groups, calculated using the Kruskal–Wallis test for continuous variables and Pearson's Chi-squared test for categorical variables

Superscript letters (a, b, c, d, e) following values for sex and diagnosis indicate post-hoc analysis results (Z-test for proportions with Bonferroni correction). Within the same row, values with different letters are statistically different (p < 0.005)

Abbreviations: *BMI* Body Mass Index, *RA* rheumatoid arthritis, *SpA* spondyloarthritis, *PsA* psoriatic arthritis, *PPAIN* Patient's Global Assessment of Pain Intensity (0–100), *PGADA* Patient's Global Assessment of Disease Activity (0–100), *BFI* Brief Fatigue Inventory (0–10), *PHQ-4* Patient Health Questionnaire-4 (0–12), Sleep Quality Score, calculated score (−4 to + 4), Social Support Score, calculated score (0–20), Physical Activity Score, calculated score (0–20), Diet Quality Score, calculated score (−5 to + 3)

### External validation of archetypes with clinical outcomes

**Fig. 2:**
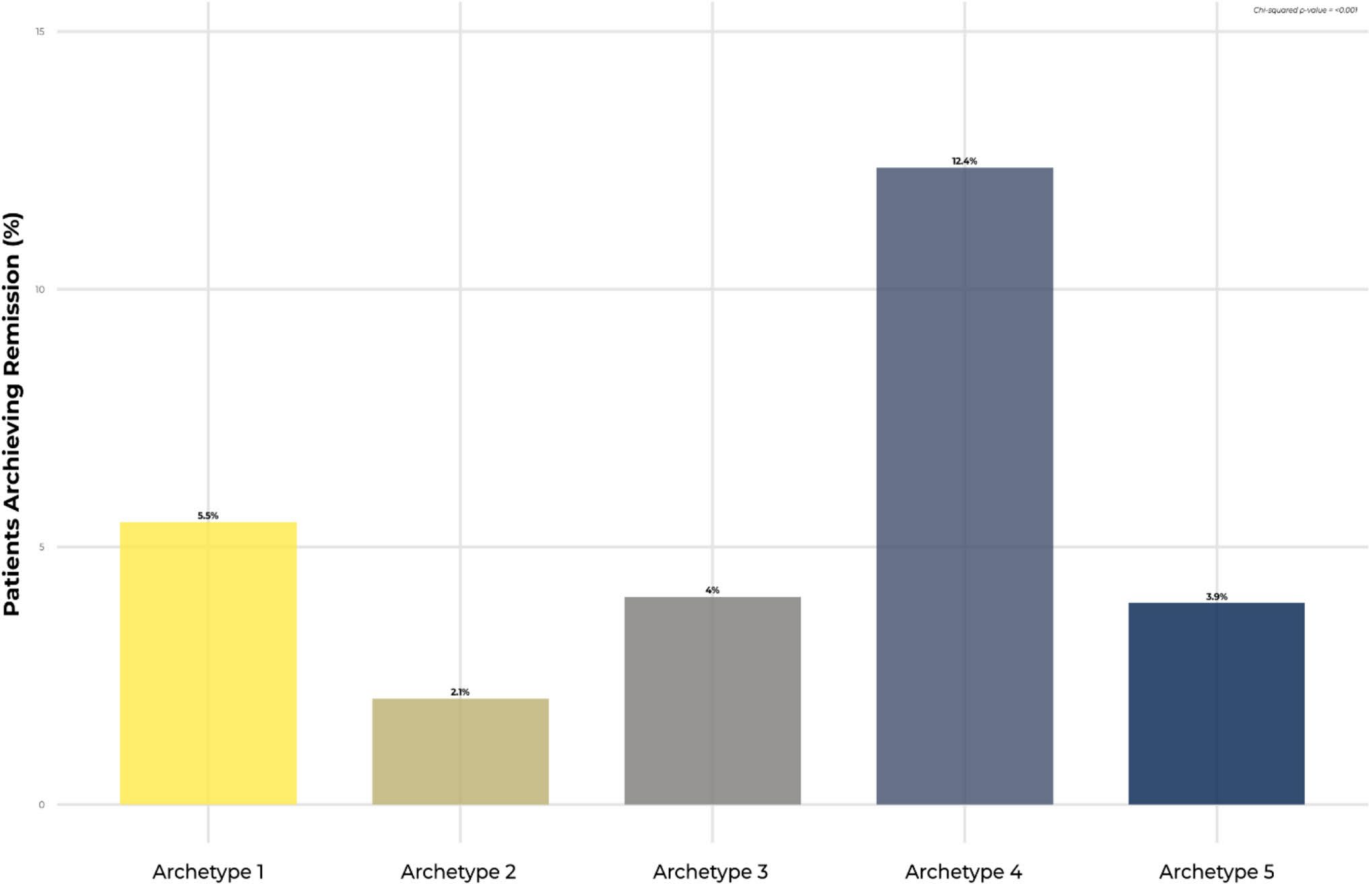
Validation. Remission achievement. Bar chart showing the percentage of patients in each archetype who achieved remission at 12 weeks. Remission was defined as a PGADA score ≤ 20 mm, restricted to baseline PGADA ≥ 40 mm. The difference between archetypes is statistically significant (χ^2^ *p* < 0.001). Archetype 4 demonstrates the highest rate of remission, while Archetypes 2 and 5 show the lowest. Error bars represent 95% confidence intervals

The five archetypes demonstrated statistically significant differences in 12-week clinical outcomes, confirming their clinical relevance. The proportion of patients achieving Remission varied significantly across clusters (χ^2^ *p* < 0.001, Fig. 2). The "High-Resilience/Low-Symptom" cluster (Cluster 4) showed the highest Remission rate at 12.4%. In stark contrast, the lowest rates were observed in the "SpA-Female, Low-Support" cluster (Cluster 2) at 2.1% and the "Psychologically Distressed, Poor Sleep" cluster (Cluster 5) at 3.9%.

**Fig. 3:**
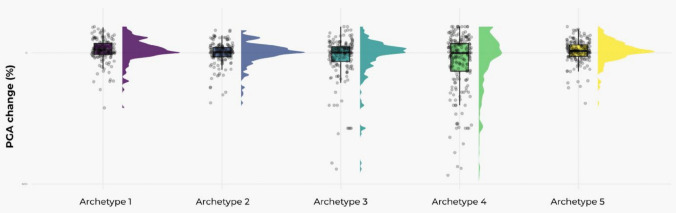
Validation. PGADA change. Raincloud plots illustrating the distribution of percentage change in Patient Global Assessment of Disease Activity (ΔPGADA%) at 12 weeks for each archetype. Each plot combines a density curve (the 'cloud'), individual data points (the 'rain'), and a boxplot. For visualization purposes, extreme outliers (ΔPGADA% < −500%) have been excluded. The overall difference between the groups is statistically significant (*p* < 0.001), with Archetype 4 showing the most favourable distribution of improvement. Note: Lower baseline PGADA limits the ceiling for % improvement (headroom effect)

Similarly, the mean percentage improvement in PGADA differed significantly across clusters (*p* < 0.001, Fig. 3). The distribution of improvement clearly favoured Cluster 4, which showed a substantial positive shift in disease activity. Conversely, Clusters 2 and 5 exhibited minimal improvement, with a considerable proportion of patients showing no change or even a worsening of their condition.

### Adjusted analyses of archetype association with outcomes

After adjusting for age, sex, diagnosis, disease duration, baseline pain, and baseline PGADA, cluster membership remained an independent factor associated with 12-week outcomes (Table 2).

**Table 2:** Adjusted associations of cluster membership with 12-week outcomes

	Outcome A: Remission		Outcome B: ΔPGADA%	
Predictor	Adjusted OR (95% CI)	*p*-value	β (95% CI)	*p*-value
**Cluster Membership (Ref: Archetype 1)**				
Archetype 2	1.04 (0.54 to 2.01)	0.902	−7.77 (−30.84 to 15.30)	0.509
Archetype 3	3.36 (1.26 to 8.96)	**0.015**	−13.42 (−56.38 to 29.54)	0.54
Archetype 4	0.49 (0.10 to 2.39)	0.376	−42.18 (−77.77 to −6.58)	**0.02**
Archetype 5	0.79 (0.31 to 1.97)	0.609	−33.59 (−59.82 to −7.37)	**0.012**
**Covariates**				
Age (years)	1.02 (1.00 to 1.03)	0.072	0.53 (−0.03 to 1.10)	0.066
Female (1 = yes)	1.41 (0.81 to 2.48)	0.228	−0.10 (−17.39 to 17.18)	0.991
Disease duration (days)	1.00 (1.00 to 1.00)	0.23	0.00 (−0.00 to 0.00)	0.678
Baseline PPAIN (0–10)	0.72 (0.61 to 0.84)	** < 0.001**	−7.06 (−11.56 to −2.56)	**0.002**
Baseline PGADA (0–10)	0.83 (0.72 to 0.96)	**0.015**	29.65 (25.06 to 34.23)	** < 0.001**
**Diagnosis (Ref: PsA)**^**1**^				
Diagnosis: RA	1.98 (0.94 to 4.16)	0.071	7.98 (−11.85 to 27.81)	0.43
Diagnosis: RA_PsA	0.00 (0.00 to inf)	1	4.86 (−36.37 to 46.09)	0.817
Diagnosis: RA_SpA	5.77 (1.14 to 29.24)	**0.034**	21.45 (−35.56 to 78.47)	0.46
Diagnosis: RA_SpA_PsA	0.00 (0.00 to inf)	0.996	−13.41 (−89.42 to 62.59)	0.729
Diagnosis: SpA	1.62 (0.70 to 3.75)	0.256	7.26 (−15.02 to 29.55)	0.523
Diagnosis: SpA_PsA	7.53 (1.90 to 29.93)	**0.004**	24.04 (−24.87 to 72.95)	0.335

^1^PsA was used as the reference category for diagnosis

Models are adjusted for all listed covariates (age, sex, diagnosis, disease duration, baseline PPAIN, and baseline PGADA). Archetype 1 serves as the reference group for cluster comparisons

**Outcome A (Remission)**: results from a logistic regression model for achieving remission at 12 weeks, defined as PGADA ≤ 20 mm (*N* = 834). Results are shown as Adjusted Odds Ratios (OR) with 95% Confidence Intervals (CI)

**Outcome B (ΔPGADA%)**: results from a linear regression model for the percentage change in PGADA from baseline to 12 weeks (*N* = 819). Results are shown as beta coefficients (β) with 95% CIs

Abbreviations: *PGADA* Patient's Global Assessment of Disease Activity, *PPAIN* Patient's Global Assessment of Pain Intensity, *RA* rheumatoid arthritis, *SpA* spondyloarthritis, *PsA* psoriatic arthritis

In the logistic regression model for Remission achievement, patients in Cluster 3 ("Male, Long-Duration") had significantly higher odds of achieving Remission compared to the reference Cluster 1 (Adjusted OR 3.36, 95% CI 1.26 to 8.96, *p* = 0.015). Higher baseline pain and PGADA were independently associated with lower odds of achieving Remission.

In the linear regression model for ΔPGADA%, Cluster 4 ("Low-Symptom/High-Resilience") and Cluster 5 ("Psychologically Distressed") showed significantly lower percentages of PGADA improvement than the reference cluster (β −42.18 and −33.59, respectively). This finding is primarily explained by the strong positive effect of baseline PGADA on this outcome (β 29.65, *p* < 0.001), indicating that patients with higher initial scores had greater potential for percentage-based improvement. Higher baseline pain was independently associated with less improvement.

## Discussion

Our analysis suggests that these data-driven patient archetypes may have clinical relevance, as they were associated with 12-week outcomes. However, given the observational design and exploratory nature of unsupervised clustering, these findings should be interpreted as hypothesis-generating rather than as evidence of definitive prognostic or causal phenotypes. This aligns with the ongoing paradigm shift in rheumatology away from generic treatment protocols and toward stratified, personalized care models driven by advanced data analytics [5, 6]. The convergence of internal stability diagnostics, which favoured a five-cluster solution, and the strong external validity of these archetypes support their potential utility for patient stratification in both clinical research and practice.

Importantly, the identified archetypes should be interpreted as statistical groupings derived from the selected baseline clinical, psychosocial, and behavioral variables rather than as definitive biological or clinical phenotypes. Although their interpretability and association with short-term outcomes support the face validity of the segmentation, external validation in independent cohorts and prospective studies will be required before these archetypes can be considered for clinical decision-making or intervention allocation.

The central finding of our study is that baseline clinical-psychosocial profiles are powerfully associated with short-term outcomes in patients using a digital therapeutic intervention. This finding directly addresses the well-documented heterogeneity in treatment response that complicates the management of RA, PsA, and SpA [1, 4]. We observed a transparent gradient of response, with the "Low-Symptom/High-Resilience" archetype (Cluster 4) achieving the most favourable outcomes. In contrast, the "Psychologically Distressed" (Cluster 5) and "SpA- High-Symptom, Low-Support" (Cluster 2) profiles exhibited the poorest outcomes. This indicates that a patient's holistic profile, encompassing psychological and social domains, may be a more potent determinant of their clinical trajectory than their specific IA diagnosis alone, a notion supported by the literature, which highlights that traditional diagnostic categories often encompass diverse patient subpopulations [1]. Our adjusted models further reinforce this, confirming that these associations persist even after accounting for baseline symptom severity and diagnosis.

From a methodological perspective, an important design choice was to exclude baseline disease activity from the clustering feature set and instead use PGADA-based remission as an external validator. This avoided measurement coupling between predictors and outcomes and ensured that the identified archetypes primarily reflected psychosocial–behavioral patterns rather than simple gradients of symptom severity. To evaluate whether an uncontrolled inflammatory burden could distort the clustering structure, particularly in the absence of medication and treatment-change data, we conducted an internal robustness check by incorporating baseline disease activity into the feature space. As expected, this alternative model produced severity-dominated clusters and therefore addressed a different research question; consequently, it was not pursued further beyond a descriptive summary in the Supplement (Table S1).

Our results strongly underscore the detrimental impact of psychological burden on clinical outcomes, a finding consistent with a vast body of literature on the interplay between psycho-neuro-immunology and rheumatic diseases [11, 12, 36]. The "Psychologically Distressed" archetype (Cluster 5), defined by severe psychological distress and poor sleep, experienced among the worst outcomes. This provides real-world evidence for the concept that psychological distress, including depression and anxiety, is linked to higher disease activity, increased pain, and blunted treatment response [12]. The mechanisms are likely bidirectional, with chronic inflammation contributing to mood disorders and psychological stress exacerbating the immune response [11, 12]. Thus, a high psychological burden appears to function as a critical barrier, limiting the potential benefits of therapeutic interventions [12].

Conversely, our findings highlight that patient resilience factors are key moderators of treatment success. The "Low-Symptom/High-Resilience" archetype (Cluster 4) was characterized by better sleep quality, stronger social support, and lower psychological distress, and demonstrated the highest remission rates. Because percentage change depends on baseline, clusters with lower baseline PGADA (e.g., Cluster 4) can exhibit smaller adjusted ΔPGADA% despite favourable clinical improvement distributions. Accordingly, we emphasize the binary remission endpoint and the overall distributional shift as the more decision-relevant validators of cluster performance. This aligns with research showing that positive psychosocial factors, such as robust social support and emotional well-being, contribute significantly to improved quality of life and treatment outcomes in patients on biologic therapies [11, 16]. Furthermore, the link between poor sleep and adverse outcomes seen in our distressed clusters is well-documented, with insomnia being a significant risk factor for disability and being closely correlated with pain and depression [14, 15]. These behavioral domains (sleep, social support, and distress management) emerge from our analysis as potentially modifiable levers that can augment standard pharmacologic care, echoing the call for more patient-centric and holistic management strategies [1, 21].

Furthermore, our findings yield essential insights into the role of physical activity. Within the "Psychologically Distressed" profile (Cluster 5), higher baseline physical activity did not translate into better outcomes, coexisting instead with poor sleep and high distress. This observation supports the emerging concept of a "physical activity paradox”, where the well-established benefits of exercise may be negated in individuals experiencing high psychological or physiological stress [19, 20]. It suggests that, for psychologically burdened patients, the quality of physical activity and the adequacy of subsequent recovery may be more consequential than sheer activity volume alone. Therapeutic programs, therefore, should prioritize not just activity prescription but also strategies to manage stress and improve recovery to unlock the full benefits of exercise.

Methodologically, this study contributes to the growing application of AI and machine learning in rheumatology to dissect patient heterogeneity [6, 7]. While prior studies have successfully used clustering to identify phenotypes based on molecular, genetic, or clinical trial data [5, 8, 9], our approach is novel in integrating a broad set of real-world, patient-reported clinical, psychosocial, and lifestyle data collected via a digital health platform. This data-driven strategy, leveraging real-world evidence captured through digital tools, exemplifies a modern approach to understanding disease complexity and advancing personalized medicine [10, 22]. Interventions using such digital tools have been shown to improve clinical outcomes and quality of life in real-world pilot studies and even in randomized controlled trials [23]. It aligns with the current vision for rheumatology in the digital era, which emphasizes the need to move beyond simple data collection towards meaningful patient stratification [37]. However, the effectiveness of such platforms is often limited by variable patient engagement, making the identification of distinct user phenotypes, as we have done here, a critical step for implementation [38].

This study has several limitations that should be acknowledged. First, its retrospective design relies on data collected through a digital application, which may introduce selection bias, as users of such technologies may differ from the general patient population [10]. Indeed, our analysis of this cohort revealed that sustained 12-week adherence is higher among older patients with longer disease duration, suggesting that younger, newly diagnosed patients may be underrepresented in long-term follow-up data. This highlights the need for targeted strategies to improve engagement in these specific subgroups. Second, a significant portion of data for some variables was missing, necessitating imputation, which could have influenced the results. Third, the follow-up period was limited to 12 weeks, which may not capture the full long-term impact of the intervention. Fourth, the absence of data on medication exposure and treatment changes limits the interpretation of differences in remission across clusters, as treatment adjustments may have contributed to outcome variability. To gauge the potential influence of uncontrolled inflammatory burden, we conducted a brief robustness check adding baseline disease activity to the feature space; this produced severity-driven clusters and was not pursued further. Residual confounding by treatment cannot be excluded. Finally, the cohort was predominantly from a single country, and further research is needed to validate these archetypes in more diverse populations.

## Conclusion

In this unsupervised segmentation study of a large, real-world cohort of patients with inflammatory arthritis, we found that cluster-level associations, independent of diagnosis and baseline severity, show that patient resilience factors (better sleep, stronger social support, lower distress) are linked to superior short-term outcomes, whereas psychological burden and weak support blunt treatment response. This suggests that diagnosis alone does not fully explain outcome heterogeneity and that behavioral recovery domains, such as sleep, social support, and distress management, are modifiable levers that can augment pharmacologic care. Furthermore, our findings suggest that therapeutic programs should prioritize the quality of physical activity and recovery, not merely activity volume, especially for patients with high levels of psychological distress and poor sleep. These insights underscore the need to move toward a more stratified and holistic model of care, where the integration of targeted behavioral interventions is key to unlocking better, patient-centered outcomes.

## Supplementary Information

Below is the link to the electronic supplementary material.

Supplementary file1 (DOCX 26743 KB)

## Data Availability

Analysis was conducted on anonymized data, under a data-processing agreement, which allowed the authors to maintain independent analytic control. Data Sharing: Midaia GmbH data is not approved for sharing.

## Declarations

### Prior presentation and abstract publication

NA.
