# Supplementary material for: Clinical–psychosocial archetypes predict short-term outcomes in inflammatory arthritis: an unsupervised segmentation study
Source: Clin Rheumatol. 2026 May 28;45(7):3943–55. doi: 10.1007/s10067-026-08186-9 (PMC13342302; doi:10.1007/s10067-026-08186-9)
Supplement: Supplementary file 1 — Supplementary file1 (DOCX 26743 KB) [file 10067_2026_8186_MOESM1_ESM.docx]

**Supplementary Table S1. Sensitivity check: descriptive characteristics of clusters obtained in an alternative model, including baseline disease activity**

| **Variable** | **Cluster A (n=737)** | **Cluster B (n=620)** | **Cluster C (n=600)** | **Cluster D (n=253)** | **Cluster E (n=247)** |
| --- | --- | --- | --- | --- | --- |
| Age, years | 42.8 ± 12.3 | 44.9 ± 12.3 | 42.7 ± 12 | 59.4 ± 12.8 | 48.4 ± 12.7 |
| Disease duration, days | 3,498 ± 3,544 | 3,622 ± 3,651 | 4,219 ± 4,167 | 8,792 ± 7,063 | 4,886 ± 4,543 |
| PPAIN (0–100) | 65.0 ± 20.1 | 23.3 ± 19.5 | 68.0 ± 21.0 | 52.6 ± 22.5 | 63.4 ± 18.9 |
| PGADA (0–100) | 71.9 ± 20.6 | 24.1 ± 18.9 | 72.8 ± 20.6 | 57.3 ± 23.1 | 69.1 ± 20.0 |
| PHQ-4 (0–12) | 2.5 ± 2.5 | 2.7 ± 2.5 | 7.0 ± 2.6 | 4.2 ± 2.8 | 4.2 ± 2.5 |
| Fatigue (BFI 0–10) | 5.3 ± 2.4 | 3.4 ± 2.2 | 6.7 ± 2.0 | 4.9 ± 2.2 | 5.9 ± 2.1 |
| Sleep Quality Score | –1.4 ± 1.9 | –1.3 ± 1.9 | –2.5 ± 1.7 | –2.0 ± 1.9 | –2.0 ± 1.7 |
| Social Support (0–20) | 15.8 ± 3.6 | 14.3 ± 4.3 | 10.2 ± 4.4 | 13.3 ± 4.5 | 11.9 ± 4.2 |
| Diet Quality Score | 1.0 ± 1.2 | 0.7 ± 1.3 | 0.4 ± 1.3 | 0.8 ± 1.3 | 0.8 ± 1.3 |
| Physical Activity Score | 7.8 ± 4.8 | 2.8 ± 3.2 | 3.8 ± 3.8 | 10.5 ± 4.9 | 2.9 ± 3.7 |
| BMI | 28.1 ± 7.0 | 27.0 ± 6.6 | 26.7 ± 6.8 | 27.5 ± 6.5 | 33.2 ± 7.1 |

Note: This table presents an exploratory clustering solution including baseline disease activity. As expected, the resulting clusters were primarily driven by symptom severity rather than psychosocial–behavioral characteristics; therefore, this model was not used for the final analysis.

Abbreviations: BFI, Brief Fatigue Inventory; BMI, Body Mass Index; PGADA, Patient’s Global Assessment of Disease Activity; PPAIN, Patient’s Global Assessment of Pain Intensity; PHQ-4, Patient Health Questionnaire-4.

**Supplementary Figure S1. Internal validation diagnostics for cluster number (k) selection.
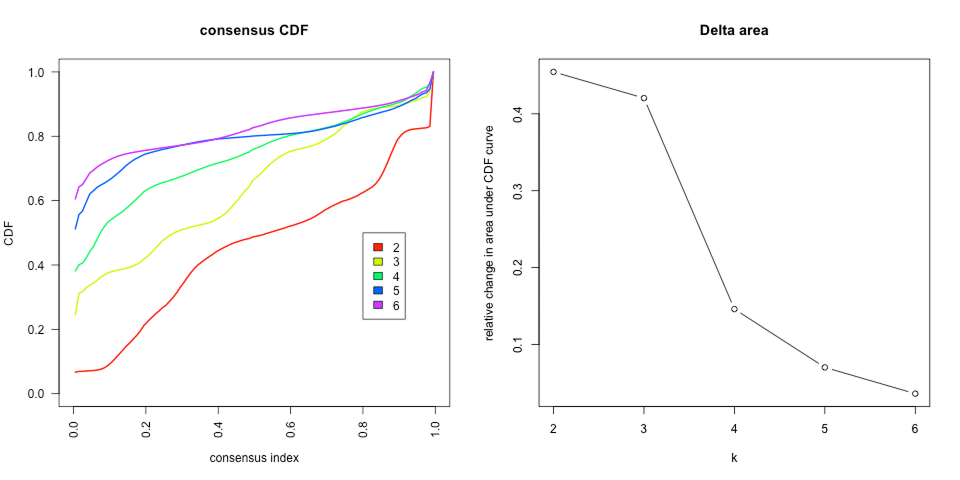
**
(A) Consensus cumulative distribution function (CDF) curves for k=2 through k=6. The curves show diminishing returns and stabilization by k=5.
(B) Delta-area plot, showing a clear 'elbow' at k=5, which indicates the point of minimal gain from adding more clusters.

**Supplementary Figure S2. Consensus matrices for k=2 to k=6.
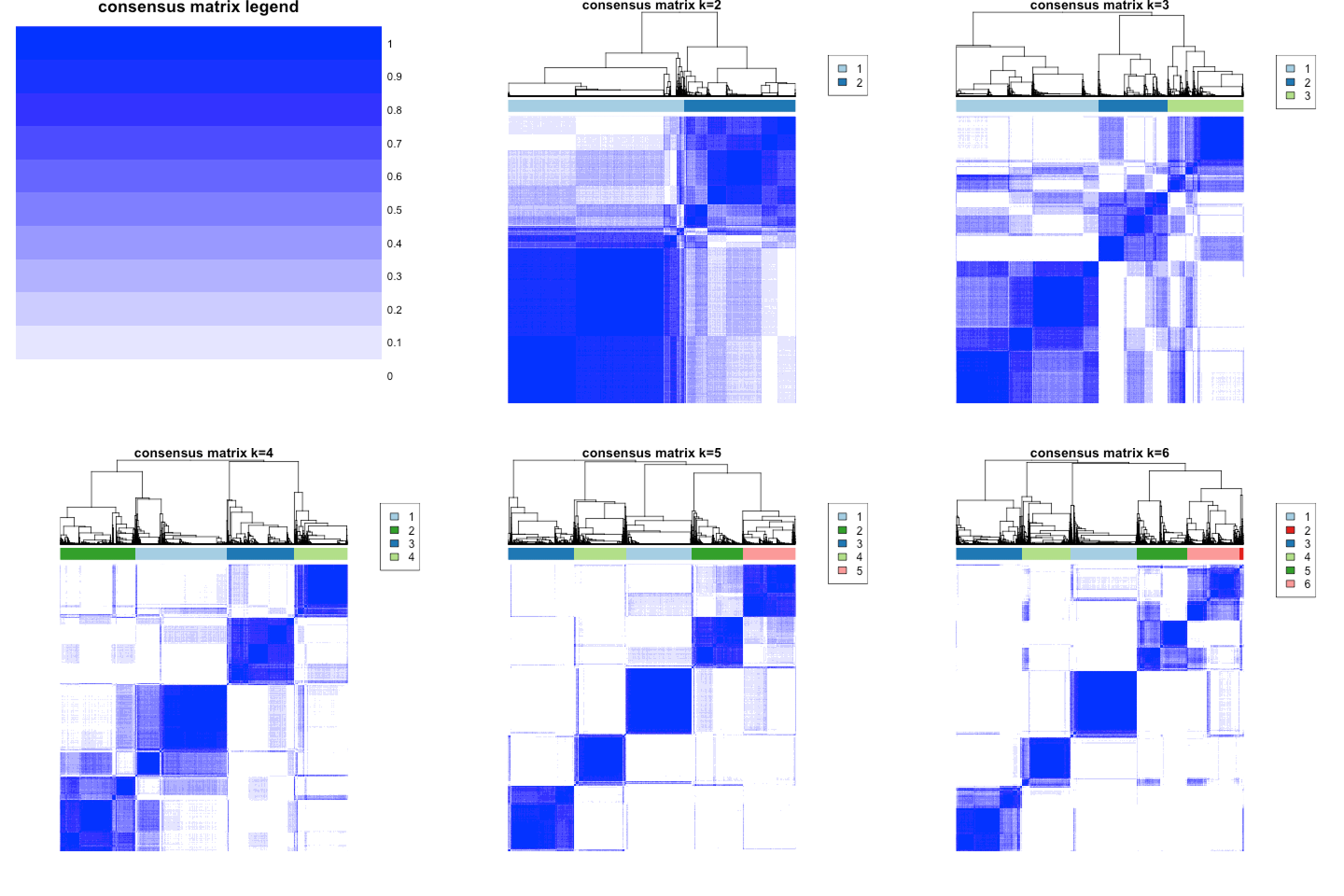
**

The matrix for k=5 shows the most stable solution, with five distinct, high-consensus (dark blue) blocks and minimal off-diagonal agreement (light colour), indicating well-separated clusters. In contrast, k=4 shows heterogeneous (less distinct) blocks, and k=6 begins to show fragmentation, suggesting over-partitioning.


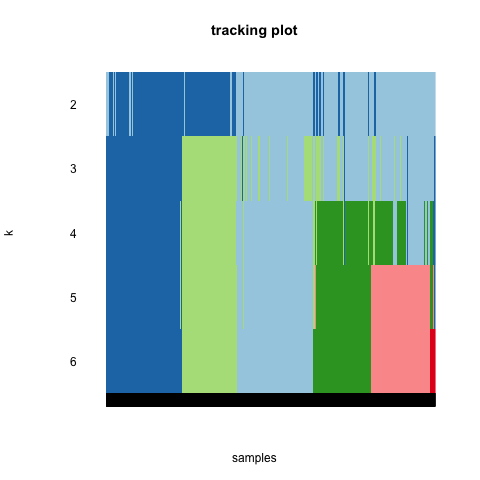
**Supplementary Figure S3. Tracking plot for k=2 to k=6.**
This plot shows how patients move between clusters as k increases. A coherent split is visible from k=4 to k=5. At k=6, small, unstable partitions begin to appear, supporting the k=5 solution.

**Supplementary Figure S4. Forest plot of adjusted odds ratios (OR) for 12-week remission.
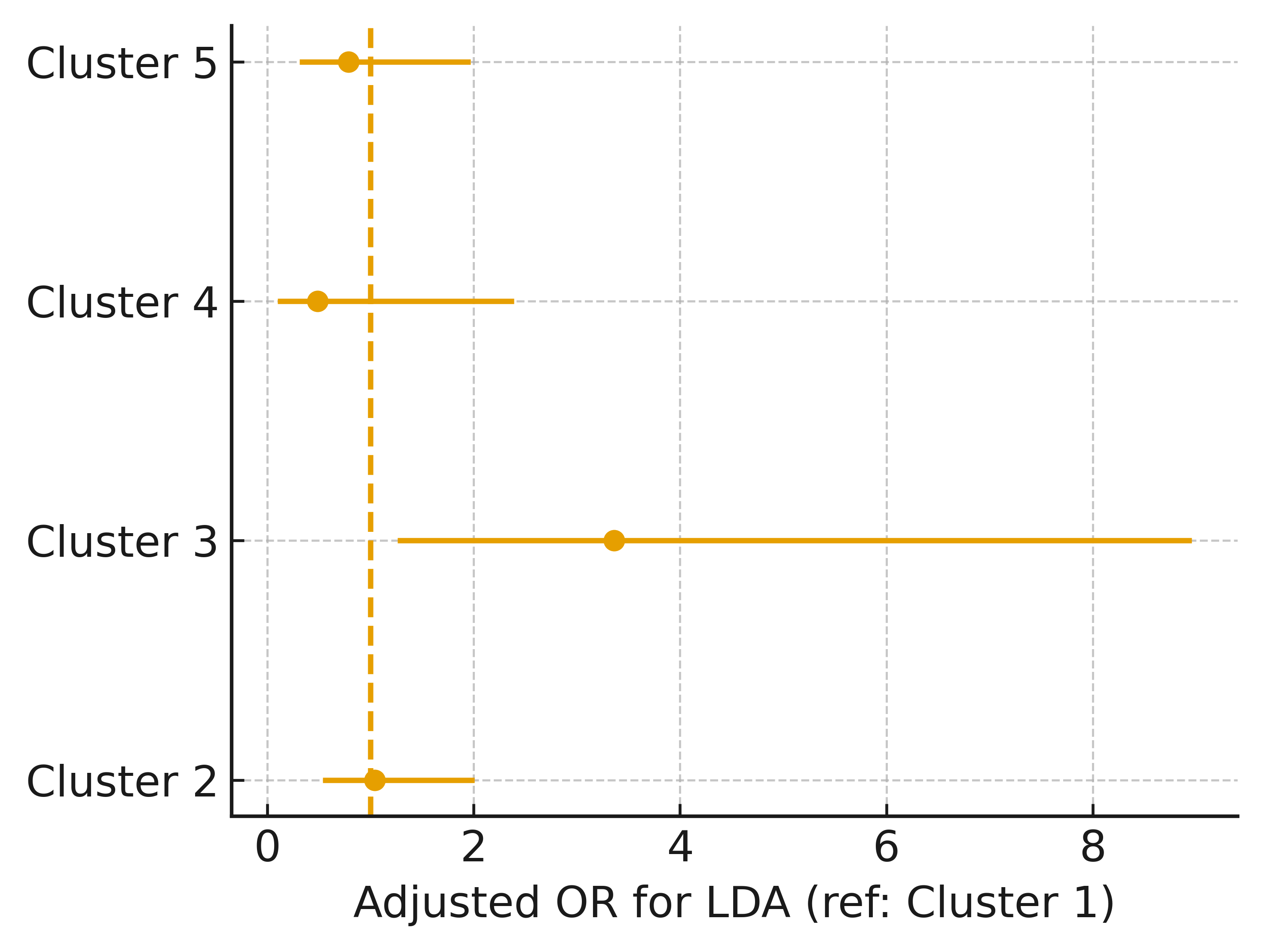
**
The model compares each archetype to Archetype 1 (reference group) and is adjusted for age, sex, diagnosis, disease duration, baseline PPAIN, and baseline PGADA. The vertical dashed line indicates an OR of 1.0 (no effect).
